# Long-term effects of on-the-job skills (mis)match on employee wellbeing and employability: a 7-wave longitudinal study

**DOI:** 10.3389/fpsyg.2025.1591769

**Published:** 2025-12-17

**Authors:** Linda Koopmans, Marieke van den Tooren, Paul Preenen

**Affiliations:** 1 Unit Health and Work, Netherlands Organization for Applied Scientific Research (TNO), Leiden, Netherlands; 2 Research Group Employability Transition, Saxion University of Applied Sciences, Enschede, Netherlands

**Keywords:** demands-abilities fit, person-job fit, skills match, skills mismatch, underqualification, overqualification, labor market, longitudinal

## Abstract

On-the-job skills match, in which employee knowledge and proficiencies (skills) are well-matched with the needs of the job, is often considered more beneficial, both for employees as well as for organizations and society, than a mismatch. A mismatch can be underqualification (when the skill level is below that which is required in the job) and overqualification (when someone has too many skills for the job they are doing). In this paper, we examined dynamics in employees’ on-the-job skills (mis)match over time, as well as the impact of these (mis)matches on employee wellbeing and employability several years later. To this end, we used 7-wave longitudinal data (2015–2021) from TNO’s Dutch Cohort Study on Sustainable Employability (CODI). The sample consisted of 7,831 Dutch employees. Descriptive data showed that about one-third (36.5%) of employees consistently reported a skills match (from T1 through T7) and 50% reported at least one change in skills (mis)match between T1 and T7 (e.g., going from underqualification to a match, despite staying in the same job with the same employer). About one in eight (12.2%) consistently reported overqualification and a small proportion of employees (0.4%) consistently reported underqualification. Predictive analyses showed that prolonged on-the-job skills match (T1–T4) affected employees’ burnout complaints, job satisfaction, labor market position and employment status 1–3 years later. Further, it was shown that prolonged skills mismatches (underqualification and overqualification) affected employees’ general health, work engagement, job satisfaction, labor market position, work ability en employment status 1–3 years later. Also, a relation was found between overqualification and burnout complaints. Main findings and their implications are discussed and recommendations for reducing on-the-job skills mismatches are made.

## Introduction

The development of employees’ knowledge and proficiencies (from now on: skills) and lifelong learning is high on the international agenda. Timely upskilling and reskilling employees are of crucial importance to ensure a good person-job fit, but also to ensure continued business performance and a sustainable and resilient labor market. For example, the Organization for Economic Co-operation and Development (OECD, 2023, 2024), the World Economic Forum (WEF) (2025) and the International Labor Organization (ILO) (2020, 2023) indicate that solving skills mismatches is essential to face challenges in the labor market and in society. The attention for skills and lifelong learning is fueled by technological developments, such as digitalization, big data and artificial intelligence. As a result, the tasks that people do in their work change or even disappear, and new tasks arise. Societal challenges also make skill development necessary. Effective utilization of human capital is necessary for, for example, the realization of the energy transition and the green economy. Demographic challenges, such as an aging population and personnel shortages, increase the need for qualified personnel even further. All these developments cause job demands to change and as a result, cause mismatches to arise between the work that needs to be done and the skills that people have to effectively carry out that work. The aim of this study is to gain more insight into how skills matches and mismatches develop within persons over time, and investigate the long-term effects of skills matches and mismatches on employee wellbeing and employability.

### Theoretical framework: person-job fit

We use person-job fit (as part of person-environment fit theory) as a theoretical framework for our study. Person-job fit refers to the fit between what the work requires and offers and what the employee can do and wants to do (Kristof, 1996). Fit theory posits that greater congruence between the abilities of the worker and the demands of the job will lead to more positive outcomes (e.g., Edwards, 1991; Kristof-Brown et al., 2005). In research, a good person-job fit has been positively related to employee wellbeing and employability (e.g., Kristof-Brown et al., 2005; Tims et al., 2016) and is of crucial importance for business performance and competitiveness and for a flourishing labor market and economy (International Labor Organization (ILO), 2020; OECD, 2023; Brunello and Wruuck, 2021). For example, a meta-analysis by Kristof-Brown et al. (2005) showed that a good person-job fit has a strong positive relationship with job satisfaction, organizational commitment, co-worker and supervisor satisfaction with the employee, job performance and tenure, and a strong negative relationship with intention to quit. Tims et al. (2016) showed that person–job fit was positively related to employees’ experiencing their work as meaningful. There is also some research arguing that the causal relationship between person-job fit and work engagement may be bidirectional. Research by De Beer et al. (2016) as well as by Valero and Hirschi (2016) indicated that work motivation and engagement was actually a predictor of person-job fit over time, rather than an outcome. Thus, more longitudinal research is necessary to shed light on the relationship between person-job fit and outcomes.

### Demands-abilities fit and on-the-job skills (mis)match

Person-job fit can be further divided into two types: the supplies-values fit and the demands-abilities fit (Edwards, 1991). The supplies-values fit is about the fit of work with someone’s needs and preferences (“what someone wants”). The demands-abilities fit is about the fit of the job with a person’s skills (“what a person can do”). The latter is particularly relevant considering the technological, societal and demographic challenges Europe is currently facing (Draghi, 2024). These challenges change the demands that are being placed on workers and require upskilling and reskilling of workers to keep a good fit between the demands of the job and the skills that workers have to effectively carry out that work. Skills mismatches therefore gain a lot of attention from for example the OECD (2023, 2024), World Economic Forum (WEF) (2025), and International Labor Organization (ILO) (2020, 2023). In these European reports, the term skills (mis)match is often used very broadly. It can refer to individual skills (mis)match with the job requirements, to skills gaps or skills shortages at the organizational level, or to skills mismatches at the country level (McGuinness et al., 2017; Vandeplas and Thum-Thysen, 2019).

In this article, we focus on the individual skills (mis)match with the job requirements, thus, on the demands-abilities fit. In skills literature, concepts of individual mismatch relate to the degree to which workers in firms possess skill or education levels that are above, below or poorly connected to those required within their current job. When the level of skills an employee has matches those needed in the job, this means an on-the-job skills match. When an employees’ skill level is above or below that which is required in the job, this means an on-the-job skills *mismatch* (Brun-Schammé and Rey, 2021). Two types of mismatches can be distinguished: underqualification (when the skill level is below that which is required in the job) and overqualification (when someone has too many skills for the job they are doing).

### Impact of on-the-job skills (mis)match

Based on person-job fit theory, it can be expected that a match between the skills of the worker and the demands of the job will lead to more positive outcomes, whereas a mismatch between skills of the worker and demands of the job will lead to more negative outcomes. There is quite a lot of research into the effects of underqualification and overqualification (e.g., McGuinness et al., 2017; Fregin, 2019; Vandeplas and Thum-Thysen, 2019; Erdogan and Bauer, 2021). In previous scientific studies, underqualification is associated with a variety of negative outcomes, both for the individual and for companies (e.g., Sim and Lee, 2018; Brunello and Wruuck, 2021). For instance, Sim and Lee (2018) showed that underqualification was negatively related to job satisfaction and organizational commitment. Fregin (2019), using PIAAC data from 30 European countries, also found a small negative relationship between underqualification and job satisfaction. Reviews by Vandeplas and Thum-Thysen (2019) and Brunello and Wruuck (2021) showed that underqualification had a negative association with a person’s income, job satisfaction and productivity. Thus, research on the effects of underqualification supports the expectation that a skills mismatch leads to more negative outcomes.

Research on the effects of overqualification paints a diffuse picture, and only partly supports the expectation that a mismatch leads to less favorable outcomes than a match. Whereas several cross-sectional studies indicate a positive relationship between overqualification and burnout (Chambel et al., 2021; Chaoyang et al., 2025; Xiao and Zhang, 2022), a review by Liu and Wang (2012) shows that the effects of overqualification on health, job satisfaction, job performance, innovative behavior and leaving intentions are inconsistent. For instance, overqualification does not necessarily seem to lead to poorer health and even seems to result in better job performance (Liu and Wang, 2012). Vandeplas and Thum-Thysen (2019) also find that overqualification raises employee productivity. In turn, a review by Velciu (2017) found that overqualification seems to have positive effects on productivity in the short term, but in the long term it leads to reduced job satisfaction and lower wages for overqualified workers. Fregin (2019), using the European PIAAC data, found no effect of overqualification on job satisfaction. A review by Erdogan and Bauer (2021) found that overqualification was related to lower psychological health and wellbeing, lower job satisfaction, and higher intentions to leave. They found conflicting relations with job performance, and indications that overqualification may lead to higher levels of proactivity and innovativeness. As with broader research on person-job fit, the causal direction of the relationship between overqualification and outcomes is not always clear. An article by Arvan et al. (2019) argues that overqualification does not lead to less job satisfaction, but that the relationship may work in the other direction: job dissatisfaction actually leads to perceptions of overqualification. Also, it matters whether overqualification is measured objectively or subjectively.

Thus, research into the effects of skills mismatch (especially overqualification) is unclear. Also, it is good to note that much of the research into on-the-job skills (mis)match is cross-sectional, looking at correlations between constructs at a single point in time, or short-term longitudinal studies. In a review on temporal person-environment fit research, Vleugels et al. (2023) note that most studies on person-environment fit are 2- or 3-wave studies, or daily/weekly diary studies, with small sample sizes. In addition, dynamic features of on-the-job skills (mis)match are interesting to examine. Currently, not a lot is known about within-person dynamics in skills mismatch with the job over a longer period. Do employees who stay in the same job, experience the same skills match or mismatch over time? Or is this construct dynamic? Currently, research on demands-abilities fit or on-the-job skills (mis)match measures this construct at one point in time. However, it seems likely that that when someone is in a mismatch for a longer period, the negative impact on well-being and employability will be stronger. This was shown for example with long-term exposure to workplace bullying (Vaktskjold Hamre et al., 2020), repeated exposure to long working hours (Virtanen et al., 2009), and chronic, sustained exposure to workplace stress (e.g., Mariotti, 2015). More longitudinal research, including more measurement waves and a larger sample size, can help to shed more light into the within-person dynamics of on-the-job skills (mis)match and the (causal) relationships between on-the-job skills (mis)match and positive and negative outcomes.

### The current study

In this study, we contribute to the research on on-the-job skills (mis)match by using a 7-wave longitudinal study of a large sample size, from The Netherlands. Using this data, we offer more insight into within-person dynamics of on-the-job skills match, and long-term effects of match and mismatch (both underqualification and overqualification) on employee wellbeing and employability.

**FIGURE 1:**
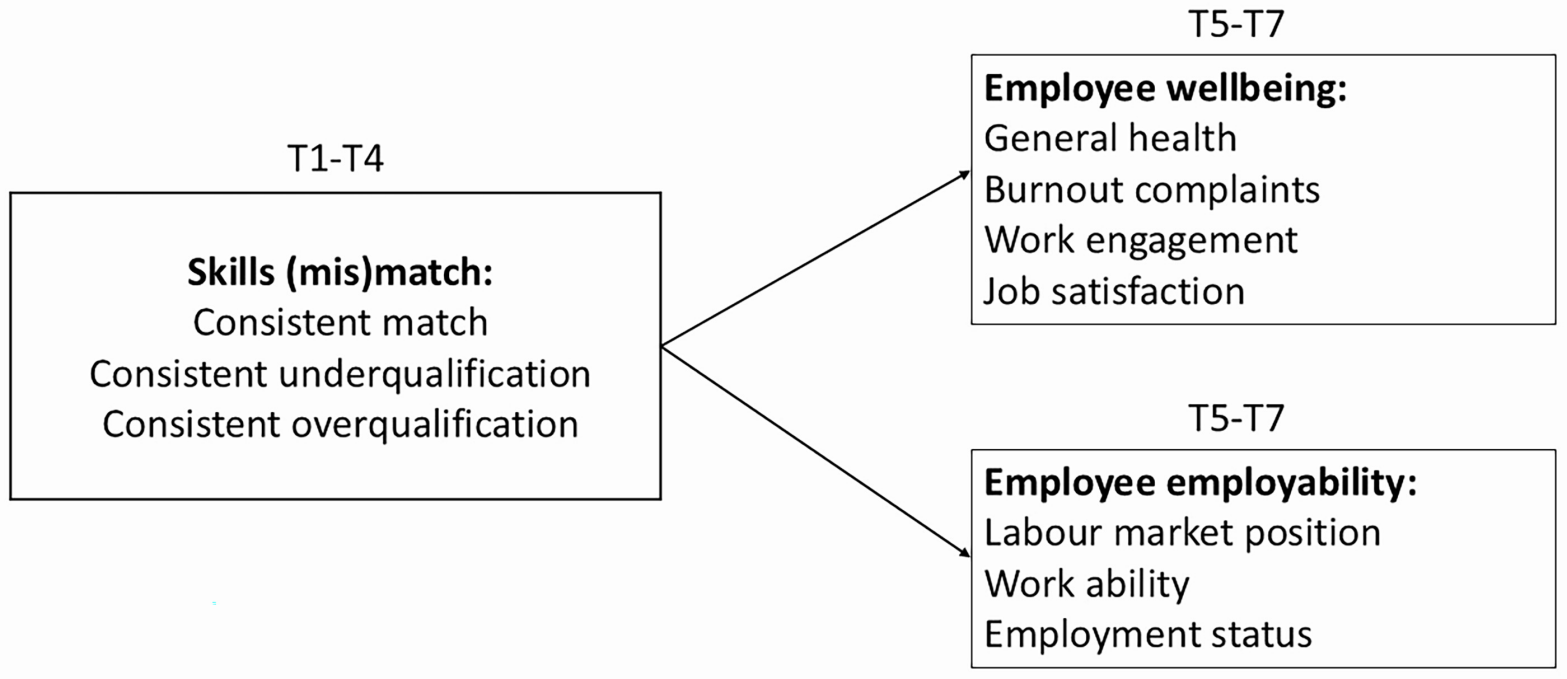
Overall conceptual model with the study framework and variables.

More specifically, we examine the impact of a 4-year “exposure” to skills match or mismatch on employee wellbeing and employability in year 5, 6, and 7. Based on person-job fit theory and previous studies (e.g., Vaktskjold Hamre et al., 2020; Mariotti, 2015), we expect that a prolonged skills match will result in more positive outcomes for employees’ wellbeing and employability, and that a prolonged skills mismatch (both underqualification and underqualification) will result in more negative outcomes for employees’ wellbeing and employability several years later. For wellbeing, the concepts of general health, burnout complaints, work engagement en job satisfaction were examined. For employability, the concepts of labor market position, work ability, and employment status were examined. See Figure 1 for an overall conceptual model.

### Hypotheses

In the current paper, and based on previous literature, the following hypotheses on the long-term impact of prolonged skills match or mismatch on various outcome measures were formulated:

*H1*: Long-term skills match from T1 to T4 has a positive association with general health, work engagement and job satisfaction, and a negative association with burnout complaints at T5, T6, T7.

*H2*: Long-term underqualification from T1 to T4 has a negative association with general health, work engagement and job satisfaction, and a positive association with burnout complaints at T5, T6, T7.

*H3*: Long-term overqualification from T1 to T4 has a negative association with general health, work engagement and job satisfaction, and a positive association with burnout complaints at T5, T6, T7.

*H4*: Long-term skills match from T1 to T4 has a positive association with labor market position, work ability and employment status at T5, T6, T7.

*H5*: Long-term underqualification from T1 to T4 has a negative association with labor market position, work ability and employment status at T5, T6, T7.

*H6*: Long-term overqualification from T1 to T4 has a positive association with labor market position, work ability and employment status at T5, T6, T7.

## Materials and methods

### Research design

This study is based on longitudinal data from a nationwide cohort study in The Netherlands: The Dutch Cohort Study on Sustainable Employability (CODI) (Van den Heuvel et al., 2022). The CODI questionnaire contains a large variety of measures on sustainable employability. At the time of this study, CODI included seven annual waves of data collection. The first wave of data originates from 2015 (T1), when a large group of 42,613 employees and 4,796 self-employed workers completed the Netherlands Working Conditions Survey (NEA; TNO/CBS) and the Netherlands Survey of the Self-employed (ZEA; TNO/CBS). In 2016, 31,740 employees and 2,291 self-employed workers from these two surveys were approached to form the new CODI cohort study. A total of 18,038 participants completed the CODI questionnaire in 2016 (response rate = 53%). In 2017 (T3), 15,194 participants completed the CODI questionnaire. In 2018 (T4), 10,212 participants. In 2019 (T5), 9,949 participants. In 2020 (T6), 10,125 participants. In 2021 (T7), 9,216 respondents completed the questionnaire. The present study is based on data from seven waves, spanning from 2015 (T1) to 2021 (T7). Participants were invited to complete the questionnaire via a letter at their home address and via their e-mail address when available. A maximum of four reminders were sent to those who did not respond. From the beginning, the questionnaire was administered digitally. In 2021, the questionnaire was largely completed on the desktop or laptop (62%). In addition, 23% completed the questionnaire on their smartphone and 15% on a tablet.

### Research population

For an overview of the CODI participants’ sociodemographic descriptives at T1 (see Table 1, column 1). Around 80% of the CODI participants were employees. Of these employees, 52.7% were males and 47.3% were females. Their average age was 41.2 years with a minimum of 15 and a maximum of 74. For the descriptive statistics, CODI participants who were employed at T1–T7 were included. Also, they had to stay in the same job and at the same employer in this period, to avoid changes in skills (mis)match due to a job or employer change. Participants who were self-employed or without a job were excluded from the current study. Participants who had more than three (of the seven) measurement moments missing were also excluded, to ensure enough data points to make reliable trajectories of their skills (mis)match over time. This selection resulted in a group of 11,691 employees. For the predictive analyses, CODI participants who were employed at T1–T4, and that stayed in the same job and at the same employer for that period, were included. Participants who had more than one (of the four) measurement moments missing were excluded, to ensure enough data points to create reliable groups. This resulted in a sample of 5,495 employees. This sample consisted of 54.5% males and 45.6% females, their average age was 43.9 years with a minimum of 15 and a maximum of 74. For more sample descriptives at T1 (see Table 1, column 2). As we wanted to examine the effects of long-term “exposure” to skills match or mismatch, we made groups of employees with “a consistent underqualification,” “a consistent match” and “a consistent overqualification” from T1 to T4. For their descriptives (see Table 1, columns 3–5). As the predictive analyses required a valid score on the outcome measures at T5, T6, and T7, due to missing data the sample for the analyses (Tables 2–8) ranged between 2,908 and 3,709 employees (depending on the outcome measure and measurement moment).

**TABLE 1:** Sociodemographic descriptives of the participants at T1.

Characteristics		Regression analyses			
	Total CODI Employees at T1 *N* = 14,400	Total sample For H1-6 *N* = 5,495	Consistent underqualification group *n* = 47	Consistent match group *n* = 3,124	Consistent overqualification group *n* = 1,074
**Gender**					
Male	52.7%	54.5%	68.6%	51.0%	56.5%
Female	47.3%	45.6%	31.4%	49.0%	43.5%
**Age group**					
15–24 years	12.2%	5.3%	0%	2.8%	9.0%
25–54 years	70.1%	74.5%	84.6%	75.7%	69.9%
55–64 years	16.7%	19.4%	15.4%	21.1%	18.8%
65 + years	1.0%	0.8%	0%	0.5%	2.3%
**Highest education level**					
Primary and secondary education (EQF 2–3)	21.2%	18.6%	12.5%	17.6%	17.8%
Post-secondary (EQF 4–5)	43.6%	43.9%	47.4%	42.4%	45.9%
Higher education (EQF 6–8)	35.3%	37.5%	40.1%	40.0%	36.3%

**TABLE 2:** Types of within-person changes in skills (mis)matches between T1 and T7.

Skill trajectory	Number of respondents (n)	Percentage of respondents (%)
Consistent underqualification	50	0.4%
Consistent match	4,264	36.5%
Consistent overqualification	1,420	12.2%
From underqualification to a match	308	2.6%
From underqualification to overqualification	91	0.8%
From a match to underqualification	247	2.1%
From a match to overqualification	1,232	10.5%
From overqualification to a match	1,067	9.1%
From overqualification to underqualification	100	0.9%
Continuous changes over the years	2,912	24.9%

**TABLE 3:** Regression analyses with skills (mis)match during T1–T4 as predictor and general health at T5, T6, and T7 as outcome measure.

Variables	Outcome measure		
	General health T5 (1 year later) [1 = bad, 5 = excellent]	General health T6 (2 years later) [1 = bad, 5 = excellent]	General health T7 (3 years later) [1 = bad, 5 = excellent]
	β	β	β
Gender [0 = male, 1 = female]	–0.005	0.006	–0.007
Age [10-year categories]	–0.040**	–0.039*	–0.046**
Education level T4 [0 = low, 1 = medium, 2 = high]	0.067***	0.052***	+ 0.056***
General health T1	0.578***	0.568***	0.548***
Underqualification T1–T4	–0.028**	0.003	–0.041**
Skills match T1–T4	–0.021	0.012	0.000
Overqualification T1–T4	–0.053***	–0.020	–0.011
*Adjusted R* ^2^	35.6%	33.6%	31.8%
*N*	3.589	3.536	3.322

*p < 0.05,

**p < 0.01,

***p < 0.001.

**TABLE 4:** Regression analyses with skills (mis)match during T1–T4 as predictor and burnout symptoms at T5, T6, and T7 as outcome measure.

Variables	Outcome measure		
	Burnout symptoms T5 (1 year later) [1 = never, 7 = every day]	Burnout symptoms T6 (2 years later) [1 = never, 7 = every day]	Burnout symptoms T7 (3 years later) [1 = never, 7 = every day]
	β	β	β
Gender [0 = male, 1 = female]	0.009	0.030	–0.004
Age [10-year categories]	–0.048**	–0.075***	–0.088***
Education level T4 [0 = low, 1 = medium, 2 = high]	–0.029*	0.030*	0.015
Burnout complaints T1	0.552***	0.521***	0.478***
Underqualification T1–T4	0.077***	0.030*	0.012
Skills match T1–T4	0.014	0.014	–0.044**
Overqualification T1–T4	0.008	0.027	–0.030
*Adjusted R* ^2^	31.4%	28.4%	24.1%
*N*	3.537	3.382	3.067

*p < 0.05,

**p < 0.01,

***p < 0.001.

**TABLE 5:** Regression analyses with skills (mis)match during T1–T4 as predictor and work engagement at T5, T6, and T7 as outcome measure.

Variables	Outcome measure		
	Work engagement T5 (1 year later) [1 = low, 7 = high]	Work engagement T6 (2 years later) [1 = low, 7 = high]	Work engagement T7 (3 years later) [1 = low, 7 = high]
	β	β	β
Gender [0 = male, 1 = female]	–0.009	0.006	0.017
Age [10-year categories]	–0.013	0.039**	–0.005
Education level T4 [0 = low, 1 = medium, 2 = high]	0.024	0.032*	0.003
Work engagement T1	0.584***	0.553***	0.534***
Underqualification T1–T4	–0.066***	0.007	0.029
Skills match T1–T4	0.005	–0.012	0.008
Overqualification T1–T4	–0.040**	–0.035*	–0.018
Adjusted *R*^2^	35.4%	31.1%	28.5%
*N*	3.339	3.178	2.908

*p < 0.05,

**p < 0.01,

***p < 0.001.

**TABLE 6:** Regression analyses with skills (mis)match during T1–T4 as predictor and job satisfaction at T5, T6, and T7 as outcome measure.

Variables	Outcome measure		
	Job satisfaction T5 (1 year later) [1 = low, 5 = high]	Job satisfaction T6 (2 years later) [1 = low, 5 = high]	Job satisfaction T7 (3 years later) [1 = low, 5 = high]
	β	β	β
Gender [0 = male, 1 = female]	0.013	0.004	–0.017
Age [10-year categories]	0.012	–0.013	–0.053**
Education level T4 [0 = low, 1 = medium, 2 = high]	0.034*	0.053**	0.035*
Job satisfaction T1	0.248***	0.196 ***	0.195***
Underqualification T1–T4	–0.086***	–0.032	–0.021
Skills match T1–T4	0.039*	0.015	0.045*
Overqualification T1–T4	–0.074***	–0.053*	–0.001
Adjusted *R*^2^	8.7%	4.9%	4.6%
*N*	3.709	3.538	3.233

*p < 0.05,

**p < 0.01,

***p < 0.001.

**TABLE 7:** Regression analyses with skills (mis)match during T1–T4 as predictor and labor market position at T5, T6, and T7 as outcome measure.

Variables	Outcome measure		
	Labor market position T5 (1 year later) [1 = low, 5 = high]	Labor market position T6 (2 years later) [1 = low, 5 = high]	Labor market position T7 (3 years later) [1 = low, 5 = high]
	β	β	β
Gender [0 = male, 1 = female]	–0.057***	–0.052***	–0.057***
Age [10-year categories]	–0.272***	–0.246***	–0.241***
Education level T4 [0 = low, 1 = medium, 2 = high]	0.063***	0.080***	0.059***
Labor market position T1	0.367***	0.380***	0.357***
Underqualification T1–T4	–0.042**	–0.042**	–0.005
Skills match T1–T4	–0.030*	0.038*	0.007
Overqualification T1–T4	–0.057***	–0.005	0.065
Adjusted *R*^2^	30.2%	29.1%	25.9%
*N*	3.619	3.426	3.111

*p < 0.05,

**p < 0.01,

***p < 0.001.

**TABLE 8:** Regression analyses with skills (mis)match during T1–T4 as predictor and work ability at T5, T6, and T7 as outcome measure.

Variables	Outcome measure		
	Work ability T5 (1 year later) [1 = low, 5 = high]	Work ability T6 (2 years later) [1 = low, 5 = high]	Work ability T7 (3 years later) [1 = low, 5 = high]
	β	β	β
Gender [0 = male, 1 = female]	–0.022	–0.027	–0.008
Age [10-year categories]	–0.097***	–0.080***	–0.132***
Education level T4 [0 = low, 1 = medium, 2 = high]	0.037*	0.037*	0.075***
Work ability T1	0.315***	0.310***	0.258***
Underqualification T1–T4	–0.061***	–0.015	0.008
Skills match T1–T4	0.025	0.026	0.016
Overqualification T1–T4	–0.033	–0.006	–0.007
Adjusted *R*^2^	12.0%	10.9%	9.5%
*N*	3.420	3.383	3.243

*p < 0.05,

**p < 0.01,

***p < 0.001.

### Research variables

The following variables from the CODI study were used in the current study:

*Skills (mis)match* was measured annually between T1 and T7 with the question: “How do your knowledge and skills match your current job?” and the following response categories: (1) I have less knowledge and skills than I need for my job; (2) My knowledge and skills match well with my work, (3) I have more knowledge and skills than I need in my job. The first response category indicates underqualification, the second response category indicates a good match, and the third response category indicates overqualification. The question originates from the Netherlands Working Conditions Survey (Van Dam et al., 2022).

Wellbeing of employees:

-    *General health* was measured annually with one item: “How do you feel, overall, about your health?” on a response scale of 1 (“excellent”) to 5 (“poor”). The item originates from the SF-12 (Ware et al., 1995).
-    *Burnout complaints* were measured annually with 5 items originating from the Dutch CBS Permanent Living Situation Survey (POLS). The questions from POLS are an adaptation of items from the Utrecht Burnout Scale (UBOS) (Schaufeli and van Dierendonck, 2000). An example item is “I feel emotionally drained by my work” on a response scale of 1 (“never”) to 7 (“every day”). The five items on burnout complaints together form one scale score. The Cronbach’s alpha of the burnout complaints scale ranged between 0.80 and 0.90 at T1–T7 (Van den Heuvel et al., 2022).
-    *Work engagement* was measured annually with 6 items originating from the vitality (vigor) and dedication scale of the abbreviated version of the Utrecht Work Engagement Scale (UWES) (Schaufeli and Bakker, 2003). An example item is “At my work, I feel bursting with energy” on a response scale from 1 (“never”) to 7 (“always”). In CODI, the dimension absorption was omitted because at least one item of this scale is not always well understood (“My work thrills me”). The 6 items on engagement together form one scale score. The Cronbach’s alpha of the engagement scale ranged between 0.92 and 0.93 at T1–T7 (Van den Heuvel et al., 2022).
-    *Job satisfaction* was measured annually with one item: “Overall, to what extent are you satisfied with your work?” on a response scale of 1 (“very dissatisfied”) to 5 (“very satisfied”). The question originates from the Netherlands Working Conditions Survey (Van Dam et al., 2022).

Employability of employees:

-    *Labor market position* was measured annually with one item on the estimation of one’s own labor market position: “I could easily get a new job with another employer” on a response scale of 1 (“totally disagree”) to 5 (“totally agree”). The question was derived from the Netherlands Working Conditions Survey (Van Dam et al., 2022).
-    *Work ability* was measured annually with one item from the Work Ability Index (Ilmarinen, 1999). Workers were asked to rate the extent to which they are currently able to work both physically and mentally on a scale from 1 (“not able to work at all”) to 10 (“work ability at your best”).
-    *Employment status* was measured annually with one item asking whether one was employed (in one paid job or multiple paid jobs), self-employed, or non-employed (e.g., unemployed, incapacitated, in (pre-)retirement, attending school/training, or housewife/househusband). The question originates from the Netherlands Working Conditions Survey (Van Dam et al., 2022).

### Analyses

First, descriptive statistics were used to get a sense of how dynamic skills matches and mismatches were over time (T1–T7). Linear regression analyses were performed to answer Hypotheses 1–6. To perform the regression analyses, a skills (mis)match grouping variable (long-term underqualification, good match or overqualification) was created. Participants who consistently classified themselves as having less skills than necessary for their job on the skills (mis)match variable from T1 to T4 (with a maximum of 1 missing year), were grouped as “Long-term underqualified.” Participants who consistently classified themselves as having a good match, or more skills than necessary for their job, from T1 to T4 (with a maximum of 1 missing year), were grouped as “Long-term match” and “Long-term overqualification,” respectively. Thus, three categorical variables were created, with a “1” indicating they belonged to this group (and a “0” indicating they did not belong to this group). Each variable was entered as the independent variable in the regression analyses. Gender, age and education level and the level of the outcome variable at T1 were included as covariates, to control for their influence on the dependent variable. A separate linear regression analysis was performed for each outcome measure and time point (e.g., health 1 year later). As the study included 6 ordinal outcome measures and 3 time points, a total of 18 regression analyses were performed. In the results of the linear regression analyses, we looked at the standardized regression coefficients (Beta), the significance level (*p*-value) and the explained variance (*adjusted R*^2^). A standardized regression coefficient should be at least 0.10 to be considered relevant, according to Cohen (1988) criteria. We used this criterion in the interpretation of our data. Analysis of variance (ANOVA) were used to examine whether scores on the nominal outcome measure employment status differed significantly between the three skills groups.

## Results

### Dynamics of skills (mis)matches

Descriptive data showed that about one-third (36.5%) of employees indicated a good skills match at all completed measurement moments (T1–T7). About one in eight (12.2%) reported prolonged overqualification and a small proportion of employees (0.4%) reported prolonged underqualification. The remaining employees (50.9%) reported at least one change in skills (mis)match between T1 and T7. For instance, about one in 10 employees went from a good match to an overqualification (10.5%), or from an overqualification to a good match (9.1%). Almost a quarter (24.9%) of employees experienced several fluctuations in skills (mis)matches between T1 and T7. Table 2 reports all changes in skills (mis)matches that were observed between T1 and T7.

### Hypotheses 1, 2, and 3 (impact on employee wellbeing)

#### General health

Results in Table 3 show that underqualification during T1–T4 was negatively related to general health at T5 (*B* = –0.03, *p* < 0.05) and T7 (*B* = –0.04; *p* < 0.01), but not at T6. Overqualification during T1–T4 was also negatively related to general health at T5 (*B* = –0.05; *p* < 0.001), but not at T6 or T7. Long-term skills match had no long-term impact on general health. The model explained 31.8–35.6% of the variance in general health at T5, T6, and T7. Most of the variance in general health at T5–T7 was explained by general health at T1.

#### Burnout complaints

In Table 4, it is shown that underqualification during T1–T4 was positively related to burnout complaints at T5 (*B* = 0.08; *p* < 0.001) and T6 (*B* = 0.03; *p* < 0.05), but not at T7. A skills match during T1–T4 was negatively related to burnout complaints at T7 (*B* = –0.04; *p* < 0.01), but not at T5 or T6. Long-term overqualification had no long-term impact on burnout complaints. The model explained 24.1–31.4% of the variance in burnout complaints at T5, T6, and T7. Most of the variance was explained by burnout complaints at T1.

#### Work engagement

Table 5 shows that underqualification during T1–T4 had a negative impact on work engagement at T5 (*B* = –0.07; *p* < 0.001), but not at T6 and T7. Further, long-term overqualification was negatively related to work engagement at T6 (*B* = –0.04; *p* < 0.05), but not at T5 and T7. Long-term skills match had no long-term impact on work engagement. The model explained 28.5–35.4% of the variance in work engagement at T5, T6, and T7. Most of the variance was explained by work engagement at T1.

#### Job satisfaction

In Table 6, results show that underqualification during T1–T4 was negatively related to job satisfaction at T5 (*B* = –0.07; *p* < 0.001), but not at T6 and T7. Long-term overqualification was also negatively related to job satisfaction at T5 (*B* = –0.07; *p* < 0.001) and at T6 (*B* = –0.05; *p* < 0.05), but not at T7. A skills match during T1–T4, on the other hand, was positively related to job satisfaction at T5 (*B* = 0.04; *p* < 0.05) and T7 (*B* = 0.05; *p* < 0.05), but not at T6. The model explained 4.6–8.7% of the variance in job satisfaction at T5, T6, and T7. Most of the variance was explained by job satisfaction at T1.

### Hypotheses 4, 5, and 6 (impact on employability)

#### Labor market position

Results in Table 7 show that underqualification during T1–T4 was negatively related to labor market position at T5 (*B* = –0.04, *p* < 0.01) and T6 (*B* = –0.04; *p* < 0.01), but not at T7. Overqualification during T1–T4 was also negatively related to labor market position at T5 (*B* = –0.06; *p* < 0.001), but not at T6 or T7. Long-term skills match, on the other hand, had a negative impact on labor market position at T5 (*B* = –0.03; *p* < 0.05), a positive impact on labor market position at T6 (*B* = 0.04; *p* < 0.05), and no impact on labor market position at T7. The model explained 25.9–30.2% of the variance in labor market position at T5, T6 and T7. Most of the variance was explained by age and by labor market position at T1.

#### Work ability

Table 8 shows that long-term underqualification (*B* = –0.06; *p* < 0.001) and long-term overqualification (*B* = –0.03; *p* < 0.05) were both negatively related to work ability at T5, but not at T6 and T7. Long-term skills match had no long-term impact on work ability. The model explained 9.5–12.0% of the variance in work ability at T5, T6, and T7. Most of the variance was explained by work ability at T1.

#### Employment status

Table 9 presents the differences in employment status (employed, self-employed, non-employed) of the different groups. It shows that those with a good match from T1 to T4 remain employed more often (96.0% at T5 and 92.2% at T6) than those with underqualification (91% at T5 and 94.8% at T6) or overqualification (94.2% at T5 and 89.2% at T6). Employees with consistent underqualification or overqualification are thus more often unemployed in later years than those with a consistent skills match with the job. They don’t often become self-employed.

**TABLE 9:** Employment status (%) at T5–T7 per skills group.

Time	Employment status	Skills group		
		Underqualification T1–T4	Skills match T1–T4	Overqualification T1–T4
T5	Employed at an employer	91.0%	96.0% △	94.2%
	Self-employed	0%	0.8%	1.4%
	Non-employed	9.0%	3.2%	4.4%
T6	Employed at an employer	94.8%	92.2% △	89.2% ▽
	Self-employed	0%	1.1%	1.4%
	Non-employed	5.2%	6.7% ▽	9.4% △
T7	Employed at an employer	83.3%	87.6%	87.2%
	Self-employed	0%	1.5%	1.1%
	Non-employed	16.7%	10.9%	11.7%

Percentages are column percentages, and are tested with the Pearson χ^2^-test (horizontal comparisons). Contrast: subgroup vs. rest. ▲ and ▼: *p* < 0.05 and Cohen’s *d* ≥ 0.20. △ and ▽: *p* < 0.05 but Cohen’s *d* < 0.20.

## Discussion

A key finding of the current study is that skills (mis)matches are generally fairly dynamic over time. About half of employees in the CODI cohort (50.9%) experienced fluctuations in their skills (mis)match over a course of 7 years, despite not having changed jobs and employers. Most employees in a mismatch move to a good match over time, though, which shows that a mismatch at a certain point in your career does not mean there is no coming back from this. As long as the mismatch is temporary, this does not have to be an issue for employee or employer. The other half of employees in the CODI cohort (49.1%) showed a stable pattern in their skills (mis)match. About one in three employees (36.5%) experienced long-term skills matches and one in eight employees (12.6%) experienced long-term skills mismatch. Most employees in this latter group experienced long-term overqualification (12.2%). From a human resource and labor market perspective, the rather large group of employees experiencing consistent overqualification indicates inefficient utilization of human capital and suboptimal realization of national productivity (Velciu, 2017). A small proportion of employees (0.4%) experienced long-term underqualification. Overall, this is a small group, but at a working population level, this is quite substantial.

Other key findings are that skills matches showed small negative associations with burnout complaints and labor market position, small positive associations with job satisfaction, and a higher rate of being employed several years later. The small negative association between skills matches and labor market position does not seem in line with person-job fit theory (Kristof-Brown et al., 2005), as a positive association would be expected here. This means that people with a good skills match are less confident that they could easily get a new job with another employer. Possibly, skills matches may be more apparent in jobs where there are enough workers. Future research should further investigate this. Unexpectedly, no effects were found for skills matches and general health, work engagement and work ability. This is also not in line with person-job fit theory. Perhaps, other job factors such as workload, autonomy, and social support have stronger effects on engagement, work ability and health, overshadowing skills matching effects.

Long-term underqualification showed small negative associations with general health, work engagement, job satisfaction, labor market position and work ability, small positive associations with burnout complaints, and a lower rate of being employed several years later. Long-term overqualification showed small negative associations with general health, work engagement, job satisfaction, labor market position and work ability, and a lower rate of being employed several years later. Unexpectedly, no effects were found for burnout complaints. Perhaps long-term overqualification may have negative consequences for personal outcomes, but not necessarily for emotional exhaustion. Future research should further investigate the long-term differential effects. All in all we can conclude that hypotheses 1–5 were partly confirmed and hypothesis 6 was not confirmed.

Although the effects are relatively small (Beta < 0.10) and do not apply to all long-term measurement points, they are of great theoretical relevance and practical value (Prentice and Miller, 2016).

### Theoretical and empirical contributions

The findings are in line with previous scientific studies showing that underqualification has potentially harmful consequences. In the literature, underqualification is often associated with a variety of negative outcomes, such as lower job satisfaction and lower productivity (e.g., Sim and Lee, 2018; Brunello and Wruuck, 2021; Fregin, 2019). The same was seen in the CODI data: underqualified workers scored lower on outcomes such as general health, job satisfaction, work ability and work engagement. However, the strength of the causal relationship seems limited. In fact, the CODI data shows that people score lower on these outcome measures right from the start of the cohort in 2015, and in the predictive analyses we therefore do not see very strong relationships between skills (mis)match and outcome measures 1, 2, and 3 years later. There is some previous research questioning the causal relationship between overqualification and outcomes (Arvan et al., 2019). The findings of the current study indicate that there may other underlying factors causing more negative scores on on-the-job skills mismatch and other variables.

Previous scientific research on the effects of overqualification presents a diffuse picture (Liu and Wang, 2012; Vandeplas and Thum-Thysen, 2019; Velciu, 2017). For instance, overqualification is associated with higher job performance in the short term, but lower job satisfaction in the long term (Velciu, 2017). Other studies find no effect of overqualification on job satisfaction (Fregin, 2019). Based on the CODI data, we see some (small) negative effects of prolonged overqualification, but generally, employees with overqualification score about the same on the outcome measures as employees with a good skills match. We also do not see any substantial changes in outcome measures for employees with overqualification over time. Interestingly, we found that people with long-term underqualification or overqualification are more likely to exit the labor market in subsequent years than those with a consistently good match. This is a very relevant addition to the literature, especially considering labor market shortages in many European countries.

An initial tentative conclusion from our study is that underqualification is indeed more unfavorable than overqualification, as is sometimes mentioned in literature (e.g., McGuinness et al., 2017). Workers with overqualification actually score similar to workers with a good skills match on all outcome measures, while workers with underqualification consistently score more unfavorably on the outcome measures.

Over time, workers don’t often seem to “get stuck” in underqualification. Most workers in our dataset only experience underqualification for a short period of time and move on to a good match. In that case, a short period of underqualification is unlikely to have any (lasting) negative effects. The finding that workers regularly “fluctuate” in their skills match with their job may also be a reason for the diffuse picture found in many scientific studies (especially on overqualification). If you experience underqualification 1 year, it does not automatically mean that you will have it the next year as well. The current study shows that experienced skills match is very dynamic. This is a new contribution to the literature, and somewhat at odds with research that states that the primary driving force behind better person-job fit is job change (e.g., Vleugels et al., 2023). Our study shows that it is possible to improve the demands-abilities fit whilst staying in the same job.

In addition to previous research, two measurement moments in CODI took place during the Covid19 crisis (T6 in autumn 2020, and T7 in autumn 2021). The data showed that the underqualified group moved closer to the good-match and overqualified groups during these measurement moments, especially on the wellbeing measures. Thus, their burnout symptoms decreased, and their engagement and job satisfaction increased. In the other groups, these outcome measures remained stable during the Covid years. Thus, it seems that the Covid crisis had some beneficial effects for the underqualified workers group. Perhaps they had the opportunity to change jobs during that period and move to a job that better suited them (e.g., moving from hospitality industry to healthcare). Or being restricted to work (remotely) has allowed them to get more mental rest.

### Limitations and recommendations for future research

A strength of the current study is the large and representative sample and the unique 7-wave study design. This is an addition to existing literature that mostly examines on-the-job skills match cross-sectionally or short-term longitudinally (Vleugels et al., 2023). Nevertheless, the present study, like any study, has several limitations we would like to address to encourage future research on this important topic. First, although inherent to a 7-wave, 7 year longitudinal study, many respondents did not complete all questionnaires. This means that the sample does not consistently represent the same group over the years. Statements about the impact of skills (mis)matches on wellbeing and employability over the years therefore do not always relate to the same individuals. Future research should try to lower the attrition of participants. Second, the skills (mis)match and outcome variables in CODI are self-report measures, in which people can overestimate themselves (Brunello and Wruuck, 2021). The group of underqualified people might therefore be larger than the numbers in this study show, as people would not readily describe themselves as underqualified. And the group of overqualified people might be smaller than can be concluded based on this study. The results may also be prone to common method bias (Podsakoff et al., 2003). Ultimately, individuals themselves might be the best judge of their own skills (mis)matches. Considerable evidence exists that perceptual measures of work-related variables do reflect the objective work environment (Spector, 1992). Therefore, we believe that the self-reports may not have seriously decreased the validity of the findings. Nevertheless, future studies could use multilevel designs and include peer or managerial judgments of employees’ skill (mis)matches, and could include more objective indicators and measures for wellbeing and employability (e.g., through test scores, participation in studies and courses). Third, we used single-item measures for general health and job satisfaction. Although this is quite common and sufficient in comparison to multiple item measures (e.g., Gardner et al., 1998), future research should attempt to replicate our results using questionnaires and with a larger number of items to enhance measurement precision.

Fourth, we should be careful with making statements about causality. We see that, from the start of CODI, employees with a consistent underqualification (from 2015 to 2018) score lower on wellbeing (general health, burnout symptoms, engagement, job satisfaction) and employability (work ability, labor market position, performance) than employees with a consistently good match or overqualification.

For the future, analyzing the CODI longitudinal data with techniques such as growth curve modeling, cross-lagged panel models or latent transition analysis could shed more light on causal relationships and changes within individuals over time. Other, more qualitative studies would also be interesting to complement quantitative studies on skills match and mismatch. Think for example of controlled, longitudinal experimental settings or longitudinal diary studies. Does underqualification lead to worse wellbeing and employability over time? Or do people with low employability automatically end up in jobs for which they are underqualified? Or do they interpret it this way because their wellbeing and employability are limited and they therefore rate their own competences lower? In addition, it is relevant to investigate which factors moderate the transition from a skills mismatch to a skills match, and vice versa. For instance, what role do opportunities for (in)formal learning, a proactive learning orientation and managerial support play? One might expect that when employees are offered sufficient opportunities for (informal) learning at work, for instance, they move more easily from a underqualification to a good match. It would be interesting to investigate which factors contribute to the shift toward a good match.

### Practical implications

Skills matches of employees seem dynamic and changeable. This is positive, because it indicates that skills mismatches can be influenced. By the person themselves, and/or by actions of the employer. Actions aimed at lifelong learning and career development can help improve skills match. For management and HRM professionals, for example, this is important to know. Also, it may make hiring and recruitment efforts easier, as a good skills match with the job can be developed over time with appropriate guidance. Our findings indicate that it is important that employees do not remain in an under- or overqualified position for too long. Although this group is not large, there still is a group of workers who remain in underqualification for a longer period (i.e., years). This group does not seem to get out of underqualification independently, and should receive support and encouragement from the employer and policy makers to achieve a good match. Developing job-related skills and competencies through both formal and informal learning is crucial, and should be fostered by managers and HR professionals in the company. Additionally, employers and HRM could invest in stimulating career competencies and proactive skills that empower employees on the labor market. These can be shaped to some extent by focused interventions and training (e.g., Akkermans et al., 2014; Koen et al., 2012). Also, employers could, if possible, think of redesigning (parts of) jobs and tasks that better fit underqualified personnel.

Employees who are in overqualification for a long time also deserve attention. In addition to the negative effects found in our study, other studies also suggest that a lack of job challenge is negatively related to learning-on-the-job (e.g., DeRue and Wellman, 2009; Dragoni et al., 2009), skill utilization (Preenen et al., 2019) and organizational commitment (e.g., Dixon et al., 2005), and positively to turnover intentions (e.g., Preenen et al., 2011). Again, employers could think of redesigning (parts of) jobs and tasks that better fit underqualified personnel. In this case, for example, by providing employees with more job challenge by enriching jobs through adding more difficulty, novelty, job autonomy and task variation (Preenen et al., 2019). Ideally, this is done in such a way that both employees and organizations benefit. Additionally, employees may be stimulated to perform extra-role behaviors that go beyond their formal job requirements (Bateman and Organ, 1983), if formal job requirements are hard to adjust. Activities could be helping and coaching coworkers, performing maintenance, solving problems at work, or replacing supervisors. From a broader labor market perspective, this group forms a potential source of knowledge and skills that should not be wasted and “used” in the best and/or better, especially in time of (future) labor shortages in many countries.

Ideally, it is better to prevent skills mismatches than to cure them. This starts during the recruitment and selection process of employees (e.g., by drawing up an appropriate skills profile, writing good job ads and a careful selection processes), and continues during the job (e.g., by providing opportunities for informal and formal learning). At the macro level a better connection between education and business is crucial, and the facilitation of intra- and intersectoral mobility, for instance through a “skills-based labor market” in which skills are central for job matching (e.g., Jooss et al., 2023).

## Conclusion

The current research shows that skills (mis)matches with one’s job are dynamic, and that a mismatch once does not mean a mismatch forever. However, a small proportion of workers do structurally experience under- or overqualification in their job, with negative consequences. In particular, workers who feel underqualified for their job experience lower wellbeing and lower employability over the years. They also exit the workforce earlier. Overqualification seems to be a problem especially at the employer and labor market level. After all, there is untapped potential. In short, it is important to take care that employees do not remain in under- or overqualification for extended periods of time. This is a responsibility at both individual, company and societal level. Individuals have a responsibility to be at the steering wheel of their own careers (King, 2004; Sullivan, 1999). Yet, actions, programs and policies aimed at lifelong learning and career development are needed to improve on-the-job skills matches, and to ultimately prevent negative consequences for workers, employers and the labor market as a whole.

## Data Availability

Publicly available datasets were analyzed in this study. This data can be found at: https://www.cbs.nl/nl-nl/onze-diensten/maatwerk-en-microdata/microdata-zelf-onderzoek-doen /microdatabestanden/codi-cohort-onderzoek-duurzame-inzetbaar heid-2016-2023.
